# Drug-related problems in hospitalized patients with type 2 diabetes mellitus: A systematic review

**DOI:** 10.1016/j.rcsop.2023.100348

**Published:** 2023-10-12

**Authors:** Mohammad Hisyamuddin Awang Jihadi, Ana Yuda, Anila Impian Sukorini, Andi Hermansyah, Naeem Shafqat, Ching Siang Tan, Long Chiau Ming

**Affiliations:** a Pengiran Anak Puteri Rashidah Sa'adatul Bolkiah Institute of Health Sciences, Universiti Brunei Darussalam, Gadong, Brunei Darussalam; b Department of Pharmacy Practice, Faculty of Pharmacy, Universitas Airlangga, Surabaya, Indonesia; c School of Pharmacy, KPJ Healthcare University, Nilai, Malaysia; d School of Medical and Life Sciences, Sunway University, Sunway City, Malaysia

**Keywords:** Patient safety, Drug safety, Diabetes, Health care quality, Health outcomes, Medical care, Pharmaceutical care, Adverse drug reaction

## Abstract

**Introduction:**

Type 2 diabetes mellitus (T2DM) is one of the non-communicable diseases which continues to rise in prevalence and mortality rate throughout the years. Drug-related problems (DRPs) are more prevalent among T2DM patients especially those with co-morbidities.

**Objective:**

The objective of this study was to review and assess the prevalence and characteristics of DRPs among hospitalized type 2 diabetes mellitus patients.

**Methods:**

The systematic review of the literature was carried out using five online databases: PubMed, Scopus, Google Scholar, Web of Science, and Cochrane Library from the inception of the database until June 2022. Studies included in the review were published in English or Malay language. The data were extracted and assessed using the Joanna Briggs Institute (JBI) critical appraisal tools.

**Results:**

A total of 939 studies were identified with 20 studies that met inclusion criteria and were included in this systematic review. The overall prevalence of DRPs in all 20 studies ranged from 7% to 94%. The most common DRPs included drug-drug interaction (DDI), adverse drug reaction (ADR), therapeutic effectiveness problems, and inappropriate medication use.

**Conclusion:**

The most common drug classes involved were antidiabetics (metformin), antihypertensives, antiplatelets and antibiotics. The risk factors contributing to DRPs included the presence of comorbidities, the number of medications, and polypharmacy. To conclude, the rate of DRPs incidence in hospitalized T2DM patients was observed to be high. Further future studies with appropriate study designs and methods of detecting DRPs will be necessary to reduce and prevent DRPs occurrences.

## Introduction

The administration of medications is essential for the treatment and prevention of health issues. Throughout the years, the number of medications developed is increasing which has led to the discovery of more complex drug regimens to treat patients. Despite the remarkable advance in drug development to treat patients, Drug-Related Problems (DRPs) may arise which can cause undesirable treatment outcomes and potentially worsen patient conditions. According to the Pharmaceutical Care Network Europe (PCNE), DRP is defined as “an event or circumstance involving drug therapy that actually or potentially interferes with desired health outcomes”.^1^ There are nine sub-categories identified causes of DRPs: 1. Improper drug selection 2. Improper dose selection 3. Inappropriate dose selection 4. Duration of treatment 5. Problem with prescribing and dispensing process 6. Issues with drug administration process 7. Patient-related including behavior 8. Patient transfer related, between primary, secondary, and tertiary care.^1^ The three main types of DRPs include Medication Error (ME) defined as “a failure in the treatment process that leads to, or has the potential to lead to, harm to the patient”.^2^^,^^3^ Adverse Drug Reactions (ADR) defined as “any response to a drug which is noxious and unintended, and which occurs at doses normally used in humans for prophylaxis, diagnosis or therapy of disease, or for the modification of physiological function”,^4^ and Adverse Event defined as “an adverse outcome that can be attributed, with some degree of probability, to an action of a drug”.^5^

The prevalence of T2DM increases with time and is globally estimated to be most common in people aged 55 to 59 years with males experiencing symptom manifestation sooner than females. In 2019, the number of adults diagnosed with T2DM was found to be the highest in China and India estimated with 116.4 million and 77.0 million, respectively. According to the IDF, the number of people with T2DM is expected to increase to 700 million globally.^6^ The first-line drug therapy for T2DM is generally metformin with several advantages including a lower risk of hypoglycemia and promoting weight loss. Depending on the patient individual glycemic needs, additional medications from different drug classes (eg., glucagon-like peptide 1 [GLP-1] receptor agonists, sodium-glucose cotransporter 2 [SGLT2] inhibitors), may be appropriate. Patients experiencing weight loss and hyperglycemia with alarming levels of blood glucose (≥300 mg/dL [16.7 mmol/L]) or A1C (>10% [86 mmol/mol]) should be considered for an early introduction to insulin therapy.7., 8, 9. Studies also confirmed that insulin therapy was associated with a higher incidence of ADR due to the hypoglycemic tendency of its use.^10^

Previous systematic reviews focused on patients with diabetes' admission to hospital due to medication problems^11^^,^^12^ with several reviews focused on patients with type 2 diabetes in an outpatient setting.^13^^,^^14^ While there are various systematic reviews focusing on the identification of DRP in patients with diabetes in a hospital setting, few systematic reviews have been conducted on the prevalence and risk factors in hospitalized patients specifically with type 2 diabetes. Therefore, the aim of this study was to review and assess literature related to DRP in adult hospitalized T2DM patients, focusing on its prevalence and risk factors.

## Methods

The Preferred Reporting Items for Systematic Review and Meta-Analysis Protocols was used for this systematic review. Five electronic databases were used for the review including PubMed, Scopus, Google Scholar, Web of Science, and Cochrane Library. The articles were searched from the inception of the database until June 2022. Various search key terms were used for each database including drug-related problem (DRP), adverse drug reaction (ADR), adverse drug event (ADE), medication error (ME), treatment related problems, medical-problem oriented plan, inpatients, and type 2 diabetes mellitus (T2DM).

Studies on DRPs or subcategories in adult (≥ 18 years) type 2 diabetic hospitalized patients of both genders were included in the review. Type 2 diabetes without or with comorbidities (eg., Hypertension, renal failure) was included in the review. Type 1 diabetes and gestational diabetes were excluded from the review. However, the search was not limited to studies that report only type 2 diabetes. If a study consists of data on DRPs for both type 1 and type 2 diabetes patients, the study was read and data on type 2 diabetes were extracted. If data on type 2 diabetes were unable to be extracted, the study was removed. A similar selection process was applied to studies that consist of data on DRPs involving type 2 diabetes in both inpatient and outpatient settings.

The type of study designs reporting data and prevalence of DRPs in type 2 diabetes hospitalized patients included cross-sectional studies, interventional studies, and cohort studies. Studies included in this review were published in English and Malay.

A software program Endnote (Chandler, AZ, United States) was used for the systematic review where the study retrieved from the database search was transferred. The software facilitates the identification and removal of duplicate articles. Two researchers first screened the articles based on the titles and abstracts. Then, the full articles were screened according to the inclusion and exclusion criteria with reasoning provided for studies that were excluded at this stage. The discrepancies between two researchers were then independently reviewed by the third researcher.

Data pertaining to study details were extracted by two reviewers. Study details include the characteristic of the study (study design, duration, setting, patient demographics), DRPs characteristics (methods to classify DRPs, most common drugs involved, overall incidence), and the risk factors for DRPs.

The methodological quality of the studies included in the systematic review was assessed by two reviewers using the Joanna Briggs Institute (JBI) critical appraisal tools. The tool uses four answers: ‘Yes’, ‘No’, ‘Unclear’ and ‘Not applicable’.

**Fig. 1:**
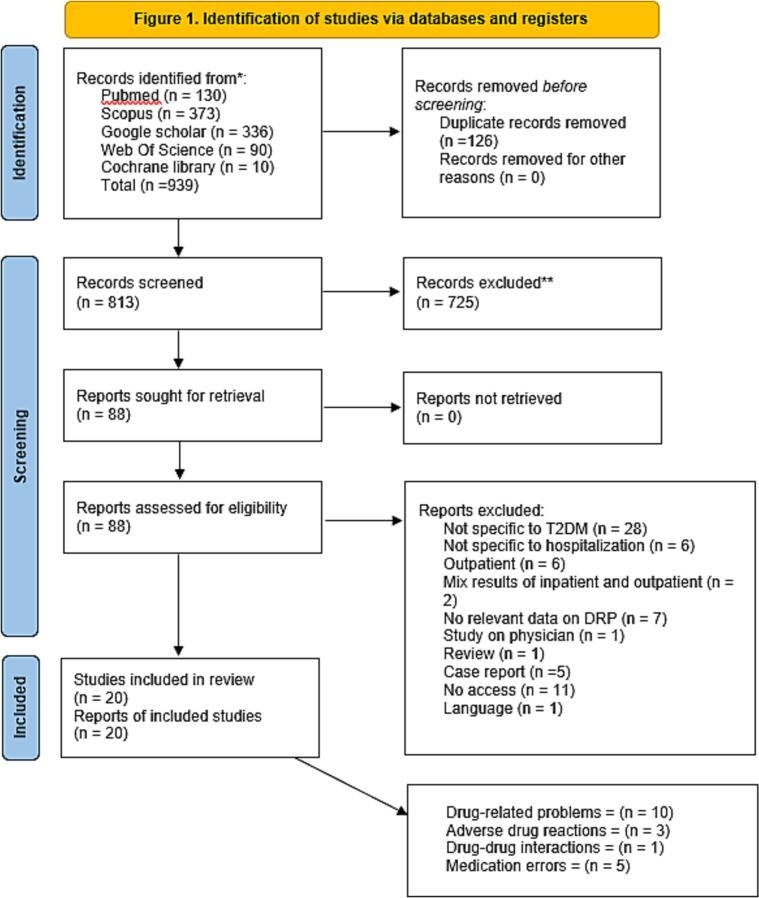
PRISMA flow chart.

A total of 939 studies were identified from the five databases which subsequently 725 studies were excluded based on the title and abstract. A total of 88 studies were screened in full text whereby 68 studies were excluded for not fulfilling the inclusion criteria. Ultimately, 20 studies met inclusion criteria and were included in this systematic review. Fig. 1 shows the Preferred Reporting Items for Systematic Reviews and Meta-Analyses (PRISMA) flow chart. Characteristics of included studies and drug related problem are presented in Table 1 and Table 2, respectively. PRISMA checklist can be found in Supplementary Information 1.

**Table 1:** Characteristics of included studies.

Author/Year and Country	Study design	Identification method	Study subjects	Duration	Setting	Patient characteristics	Outcome
Corsonello et al., 2005 ^15^ Italy	Observational study	Incident reports	Renal failure and elderly patient with diabetes	4 months	Community and university hospitals	Total 2257 Age 72.1 ± 11.4	A total of 231 (10.2% of all patients) patients having an ADR diagnosed
Varghese et al., 2007 ^16^ USA	Prospective medical record review	Chart review & Incident reports	Hospitalized patients receiving antihyperglycemic therapy”	August 16, 2004, to November 15, 2004	University hospital	Total 2174	Of the 484 hypoglycemic episodes, 46.9% occurred in patients with type 2 DM
Indriani et al., 2010 ^17^ Indonesia	Retrospective study	Chart review	T2DM patients with non-complication	January 2009 to March 2010		Total 14 Age Female 71%	DRPs drug interactions occur at 29%, ADR of 7%, 14% needed medication, and do not need drugs amounted to 7%
Okayasu et al., 2012 ^18^ Japan	Retrospective study	Incident reports & chart review	T2DM patients who initially administered metformin	September 1, 2009 to August 31, 2010	University hospital	Total 101 Age 60.7 (14–86)	The overall incidence of diarrhea observed was 26.7% (27 of 101 patients)
Kosmalski et al., 2012 ^19^ Poland		No info	T2DM patients using metformin	2009 to 2011	Department of Internal Diseases, Diabetology and Clinical Pharmacology of the Medical University of Lodz	Total 558 Age 66.65 ± 12.73 Female	275 patients were found to be contraindicated and despite this 120 of them were using this medication at an average dose of 1793.91 ± 701.61 mg. Only three patients reported moderate dyspepsia
Inamdar et al., 2016 ^20^ India	Survey study	Survey	Elderly patients with T2DM	–	Elderly in general medicine department (general ward and intensive care unit)	Total 123 Age 68.84 ± 7.33 Female 47 (38%)	364 DRPs were identified in all 123 study subjects
Herman et al., 2016 ^21^ Indonesia	Retrospective data collection	Chart review	Elderly T2DM patients	January to December of 2013	Inpatient private hospital	Total 50 Age 65.0 ± 4.46 Female 35 (70%)	15 (30%) of 50 patients identified with inappropriate use of drug by Stopp criteria
Zazuli et al., 2017 ^22^ Indonesia	Prospective study	No info	DRPs in T2DM patients with hypertension	October to December 2015	T2DM patient with HTN admitted to inpatient ward	Total 90 Age Female 74 (82.8%)	261 DRPs were identified
Hidayati et al., 2018 ^23^ Indonesia	Retrospective descriptive study	Incident reports	T2DM patients with complications of comorbidities	January to December 2016	Inpatient pharmacy depo	Total 49 Age Female 28 (57.14%)	Drug interaction of metformin with simvastatin was found in 3 people (6.12%)
Maharani et al., 2018 ^24^ Indonesia	Prospective study	Chart review & incident reporting	DRPs in hospitalized geriatric patients with diabetes mellitus	February to April 2015	Geriatric patient admitted to the army hospital	Total 26 Age > 60 Female 19 (73.1%)	166 DRPs were identified from 299 drug treatments obtained for 26 patients
Salam et al., 2018 ^25^ Indonesia	Observational study with a cross-sectional approach	Chart review	DRPs in T2DM patients with complications of macrovascular disease	July to August 2018	T2DM with macrovascular complication admitted to hospital	Total 20 Age Female 14 (70%)	100% potential problems and 35,3% problem manifestations consisting of adverse drug reactions, non-optimal treatment effects, untreated indications, and treatment unnecessary
Siregar et al., 2018 ^26^ Indonesia	Retrospective observational study	Chart review	DRPs among T2DM patients	January to June 2016	T2DM patient with HTN admitted to hospital	Total 30 Age Female 17 (56%)	DRPs in the category of drug interactions in theory was potentially 220 times (90%). There were 18 cases of drug doses too low and 18 cases of drug doses too high
Hussain et al., 2019 ^27^ India	Prospective interventional study	Chart review, direct observation (pharmacist intervention) & Interview (physician & patient)	Diabetes mellitus patients with hypertension	Six months	T2DM patient with HTN admitted to hospital	Total 122 Age 64.3 ± 1.05 Female 63 (52%)	99 patients (81%) experience drug-drug interactions
Indriani et al., 2019 ^28^ Indonesia	Retrospective cross-sectional	Chart review	T2DM patients in the ward patient	May to June 2018 (2 months)		Total 100 Age Female 58%	Most common DRPS is drug interactions that is 94.62%
Nzayisenga et al., 2019 ^29^ Africa	Retrospective cross-sectional study	Chart review	DRPs among T2DM patients with hypertention	January 2013 to December 2017	T2DM patient with HTN admitted to hospital	Total 385 Age Female 238 (61.56%)	The prevalence of DRPs was 81.29% (313/385)
Acharya et al., 2020 ^30^ India	Retrospective observational study	Chart review	T2DM patients with comorbidities	August 2019 to March 2020	Tertiary care hospital	Total 250 Age 62.5 ± 12.86 Female 72 (28.8%)	226 DRPs with an average of 0.91 DRP per patient
Inamdar et al., 2020 ^31^ India	Prospective interventional study	Chart review & direct observation (pharmacist intervention)	DRP among T2DM patients	Six months	General medicine department	Total 107 Age Female 43 (40.21%)	A total of 278 DRPs were identified after assessing 107 study
Sharma et al., 2020 ^32^ India	Cross-sectional study	Chart review	Potential Inappropriate medication use among T2DM patients	August 2019 to January 2020	Elderly in tertiary care hospital	Total 150 Age 68.85 ± 5.51 Female 68 (45,3%)	Overall, 58 (38.7%) patients were prescribed at least one PIM, 37 (24.7%) were prescribed two PIMs, and 16 (10.7%) were prescribed ≥ three PIMs
Mader et al., 2022 ^33^ Austria	Single-center survey	Questionnaire	T2DM patients with medication errors	April 2017 to February 2019	Department of Endocrinology and Diabetology	Total 100	25% of patients already suffered at least one drug error, whereby prescribing a wrong dose seemed to be the most common type of error
Nigussie et al., 2022 ^34^ Pakistan	Prospective cross-sectional study	Chart review (data abstraction form) & interview for missing files	Inappropriate antidiabetic medication therapy among T2DM patients	November 2021 to January 2022	Medical and surgical wards	Total 138 Age 58 ± 16.1 Female 80 (58%)	The overall prevalence of inappropriate anti-diabetic medication therapy was 70.3%

**Table 2:** Drug-related problem characteristics.

Author/Year and Country	Method of classify DRPs	Most common drugs	Overall incidence
Corsonello et al., 2005 ^15^ Italy	–	–	231 (10.2% of all patients) ADR identified
Varghese et al., 2007 ^16^ USA	Establish operational definitions	Specific drug class study, Antihyperglycemic (no specific drug name mentioned)	Of the 484 hypoglycemic episodes, 46.9% occurred in patients with type 2 DM,
Indriani et al., 2010 ^17^ Indonesia	Drug Information Handbook (DIH) edition 14 MIMS Indonesia edition 72,007/2008 Drug Interaction Facts (DIF) ISO Indonesia volume 442,009/2010 Informatory Obat Indonesia (IONI) 2000.	–	DRPs drug interactions occur at 29%, ADR of 7%, 14% needed medication, and do not need drugs amounted to 7%
Okayasu et al., 2012 ^18^ Japan	Common Terminology Criteria for Adverse Events (CTCAE version 4.0)	Specific drug study (metformin)	The overall incidence of diarrhea observed was 26.7% (27 of 101 patients)
Kosmalski et al., 2012 ^19^ Poland	–	Specific drug study (metformin)	275 patients were found to be contraindicated and despite this 120 of them were using this medication 3 patients reported moderate dyspepsia
Inamdar et al., 2016 ^20^ India	PCNE	Antihypertensives (108)- DDI, DWI Antidiabetics (96)- metformin + metoprolol/rabeprozol/insulin DDI /ADR Antibiotics (41) Anti-emetics (29) Antiplatelets (36)	364 DRPs were identified in all 123 study subjects.
Herman et al., 2016 ^21^ Indonesia	Start-Stopp criteria	–	15 (30%) of 50 patients identified with inappropriate use of drug
Zazuli et al., 2017 ^22^ Indonesia	American Diabetes Association. Standards of medical care in diabetes—2015 Consensus guidelines on the management and prevention of type II diabetes mellitus in Indonesia 2015 Drug Information Handbook 2011–2012. 20th	–	261 DRPs were identified
Hidayati et al., 2018 ^23^ Indonesia	Stockley 2008; Stockley 2010 Drug interaction book Drug interaction journal study on T2DM patient with hyperlipidemia	Metformin + simvastatin 3	Drug interaction of metformin with simvastatin was found in 3 people (6.12%)
Maharani et al., 2018 ^24^ Indonesia	Pharmacotherapy A Pathophysiologic Approach, 7th edition Drug Information Handbook, 17th edition Geriatric Dosage Handbook 2005 Journal of Pharmacy of Clinic MIMS ISO 2014 Micromedex PCNE	Ranitidine, omeprazole with/without sucralfate or rebamipide Antibiotics, ACE inhibitor- DDI hypo/hyperglycemia	166 DRPs were identified from 299 drug treatments obtained for 26 patients
Salam et al., 2018 ^25^ Indonesia	PCNE	–	100% potential problems and 35,3% problem manifestations consisting of adverse drug reactions, non-optimal treatment effects, untreated indications, and treatment unnecessary
Siregar et al., 2018 ^26^ Indonesia	–	–	DRPs in the category of drug interactions in theory was potentially 220 times (90%). 18 cases of drug doses too low and 18 cases of drug doses too high.
Hussain et al., 2019 ^27^ India	Helper Strand classification Lexicomp Micromedex	Aspirin, clopidogrel, warfarin, atorvastatin and some drugs acting on the CNS- DDI Antihypertensives- ADR	99 patients (81%) experience DDI
Indriani et al., 2019 ^28^ Indonesia	PCNE American Standards of Medical Care in Diabetes 2018 Drug Interaction Drug Information Handbook Indonesian Endocrinology Society	–	Most common DRPS is drug interactions that is 94.62%
Nzayisenga et al., 2019 ^29^ Africa	PCNE	Antihypertensive (270/385) Antidiabetics (267/385) Mainly drug/dose selection	The prevalence of DRPs was 81.29% (313/385)
Acharya et al., 2020 ^30^ India	Pharmaceutical Care Network Europe (PCNE) classification Helper Strand classification Micromedex Medscape Lexicomp Drugs.com	Metformin + ciprofloxacin 2 Insulin	226 DRPs with an average of 0.91 DRP per patient
Inamdar et al., 2020 ^31^ India	PCNE	Metformin + Metoprolol 64 (33.16%) Metformin + Ranitidine 51 (26.42%)	278 DRPs were identified after assessing 107 study
Sharma et al., 2020 ^32^ India	Beers Criteria 2019	Short-acting insulin Glimepiride PPI- PIM Clonazepam	58 (38.7%) patients were prescribed at least one PIM, 37 (24.7%) were prescribed two PIMs, and 16 (10.7%) were prescribed ≥ three PIMs
Mader et al., 2022 ^33^ Austria	No reference (survey study from patient perspective)	–	25% of patients already suffered at least one drug error, whereby prescribing a wrong dose seemed to be the most common type of error
Nigussie et al., 2022 ^34^ Pakistan	Cipolle's DRP classification	Biguanides (64) Insulin (61)	The overall prevalence of inappropriate anti-diabetic medication therapy was 70.3%

A wide range of scores was allocated across the 20 studies with an overall of standard quality. Three studies used an inappropriate method (purposive sampling) of recruiting participants.^21^^,^^25^^,^^26^ Individual quality assessment scoring is presented in Supplementary Information 2.

## Characteristics of studies included

Retrospective studies constituted the majority of the articles (40%), with different various observational methodological approaches (Table 1). Two prospective studies included were conducted with interventional methods implemented. Most of the studies were carried out in Asia (75%): Indonesia,^17^^,^^21^, ^22^, ^23^, ^24^, ^25^, 26.^,^^28^ India,^20^^,^^27^^,^30., 31, 32 Japan,^18^ and Pakistan,^34^ followed by Europe (15%): Italy,^15^ Poland,^19^ Austria,^33^ Africa (5%)^29^ and North America (5%).^16^ The majority of the studies were conducted in the general hospital ward with only 2 studies reported to have been conducted in the endocrinology and diabetology department.^19^^,^^33^

In all of the 20 studies, a total of 6844 patients were recruited with only adults (≥ 18 years). Of these, five studies were focused only on geriatric patients (≥ 60 years).^15^^,^^20^^,^^21^^,^^24^^,^^32^

The identification of DRPs using chart review only was utilized in eight studies.^17^^,^^21^^,^^25^^,^^26^^,^^28^, 29., 30.^,^^32^ Five studies implemented more than one method of detecting DRPs.^16^^,^^18^^,^^24^^,^^27^^,^^31^^,^^34^ Five studies used incident reports (with/without methods combination) for adverse drug reactions and drug interactions.^15^^,^^16^^,^^18^^,^^23^^,^^24^ Two studies used surveys or questionnaires as means of collecting data on DRPs from the patient.^20^^,^^33^ Two studies do not provide information on methods of detecting DRPs.^19^^,^^22^ In all of the 20 studies, a total of 27 sources of information were used to detect and classify DRPs. Eight studies used one source only.^18^^,^^20^^,^^21^^,^^25^^,^^29^^,^^31^^,^^32^^,^^34^ Seven studies used more than one source.^17^^,^^22^, ^23^, ^24^^,^^27^^,^^28^^,^^30^ One study establishes operational definitions as its source.^16^ No available sources were mentioned in four studies.^15^^,^^19^^,^^26^^,^^33^ Micromedex was the most used source of information (*n* = 3)^24^^,^^27^^,^^30^ followed by Lexicomp (*n* = 2)^27^^,^^30^ and American Diabetes Association (n = 2).^22^^,^^28^

The overall prevalence of DRPs in all 20 studies ranged from 7% to 94%. One retrospective study reported antidiabetic drug interactions only and found 6.12% of 49 patients experiencing DDIs. Three different sources were used to identify and classify the DRPs detected.

The majority of the studies investigated DRPs as a whole (*n* = 11) with no specification on the type of DRPs investigated, for example DDI, ADR, therapeutic effectiveness and dosage problems. Five of these studies identified DDIs as the main type of DRP found with a range of 29% to 94%.^17^^,^^20^^,^^27^^,^^28^^,^^31^ One retrospective study found that 37.6% of 250 patients have insufficient clinical information during the hospitalization period.^30^ The necessary clinical parameters for monitoring diabetes including blood glucose levels were not documented. Only one study reported therapeutic effectiveness problems as the common DRP with a frequency of 50.6%.^24^ The problems observed include the medication being ineffective and the failure of treatment. One study reported drug choice problems in 55.17% of 90 patients with the primary source of DRPs being drug/dose selection, which was similarly found in another study conducted by Nyazisenga and colleagues (2019). with frequencies of 62.16% and 36.97% respectively.^22^^,^^29^ No drug prescribed with clear indication has the highest frequency of 25.3% under the drug choice problem subdomain.

Two studies reported ADR only with one study focused on a specific antidiabetic drug (metformin) and another on a specific drug class (antihyperglycemic) with no drug name mentioned.^16^^,^^18^ In a study of ADR on a specific drug (metformin), the occurrence of diarrhea was found in 26.7% of 101 participants while a study of ADR on a specific drug class (antihyperglycemic) found 46.9% of 484 hypoglycemic episodes manifested in T2DM patients.

In this review, nine studies reported the most common drugs involved in DRPs, the majority of the drugs involved were antidiabetics, antihypertensives, antiplatelets and antibiotics. Four studies reported antidiabetics (metformin) to be commonly involved in DDIs.^20^^,^^23^^,^^30^^,^^31^ Inamdar and colleagues (2020) reported a common DDIs of metformin with metoprolol (33.1%) and ranitidine (26.4%).^31^ One study reported common DDIs with cardiovascular medications such as aspirin, clopidogrel, warfarin, and atorvastatin whereas antihypertensives such as hydrochlorothiazide and furosemide are associated with a higher occurrence ADRs.^27^

Five studies examined medication errors such as potentially inappropriate medication (PIM) and dosage problems.^19^^,^^21^^,^^32^, ^33^, ^34^ One of the studies investigated a specific drug (metformin) use in the elderly and observed 82% of 335 patients to be contraindicated with only three reported dyspepsias during hospital stay.^19^ Two studies investigated PIM in the elderly population with a prevalence of 30% and 74% having at least one PIM.^21^^,^^32^ Two studies reported a ME prevalence of 25% having at least one ME and 70.3% respectively with the most common problem associated with dosage problems.^33^^,^^34^ The study with a 70.3% prevalence further specifies 29.2% receiving doses too high and 27.9% doses too low.^34^ These findings aligns with another study that reported up to average 9.35 treatment related problems per patient, with 53% considered as major and 28% as moderate treatment related problems in hospitalized internal medicine patients in Jordan.^35^

Three studies investigated DRPs in specific drugs or drug classes (metformin, antihyperglycemics).^16^^,^^18^^,^^19^

Thirteen studies included the risk factors contributing to DRPs. The most prevalent risk factors for DRPs involved the presence of comorbidities found in six studies with the majority having hypertension (HTN).^20^^,^^22^^,^^27^^,^^28^^,^^31^^,^^34^ Five studies reported on age,^20^^,^^24^^,^^28^^,^^29^^,^^34^ four studies on the number of medications,^22^^,^^27^, ^28^, 29. another four studies on polypharmacy,^20^^,^^29^^,^^31^^,^^32^ and three studies on gender.^18^^,^^28^^,^^32^ The list of risk factors reported in the studies is presented in Supplementary Information 3.

## Discussion

In this systematic review, we examine the overall prevalence of DRPs in T2DM hospitalized patients with risk factors influencing DRPs and the most common drugs involved with the DRPs. In this review, ten studies investigated DRPs as a whole, followed by six studies on ME, three studies on ADRs and only one study specifically on DDIs.

The ideal outcome of treatment for T2DM patients can be defined generally as attaining the targeted glycemic control mainly by the adjustments in lifestyle together with the initiation of drug therapy. Therapeutic success can be more thoroughly defined as slowing or halting disease development and minimizing the risk factors associated with disease complications.^36^ The first line of drug therapy for diabetes is the initiation of metformin with a slow intensification of therapy depending on the disease progression with the goal of achieving targeted glycemic control as stated previously.^7^ The presence of DRPs has a potential negative impact on the outcome of diabetes treatment as well as any other conditions. The consequences of DRPs interfere with the desired clinical outcome worsening patient quality of life which emphasizes the importance of pharmaceutical care in preventing unfavorable health outcomes.^37^

One of the ways to diagnose diabetes is by using the A1C test to measure the average blood glucose for the past two to three months. A1C result showing a value of 6.5% and above indicates an individual is diagnosed with diabetes. Another test known as fasting plasma glucose (FPG) is performed during fasting when an individual is not consuming food or drinks for at least eight hours to measure the fasting blood glucose level. American Diabetes Association defines diabetes mellitus when an individual's fasting blood glucose value is 126 mg/dL and above.^38^ In this review, no studies specified the test used to diagnose patients with type 2 diabetes. Before the progression to T2DM, an individual often goes through a stage known as prediabetes in which they show a high level of blood glucose but are still not considered high enough to be identified as T2DM.^39^

The methods for identification of DRPs used in the studies include chart reviews, incident reports, surveys, direct observation, and minimal interviews. The majority of studies included in this review used chart review as the primary method for the detection of DRPs.^16^, ^17^, ^18^^,^^21^^,^^24^, ^25^, 26., 27, 28, 29., 30., 31, 32^,^^34^ The use of chart review at the prescription stage demonstrates a higher detection of DRPs with low clinical significance. The outcome of the chart review is dependent on the ability of the reviewer to detect the triggers.^40^^,^^41^ The second most used method was incident reporting.^15^^,^^16^^,^^18^^,^^23^^,^^24^ Five studies implemented more than one method of detecting DRPs with combinations of chart review with direct observation, interview, or incident reports.^16^^,^^18^^,^^24^^,^^27^^,^^31^^,^^34^

In this systematic review, a wide variety of medications was involved with DRPs in all 20 studies. Most of the studies did not specify investigating one specific drug class only however, results presented in the studies were mostly associated with class of antidiabetic medications only.

Two studies performed an investigation on a specific drug which was metformin.^18^^,^^19^ Overall, the most common antidiabetic drug classes associated with DRPs in this review were biguanide (metformin), followed by cardiovascular drugs mainly antihypertensives with several studies specifically investigating DRPs in T2DM patients with HTN as comorbidity. The higher prevalence of comorbidities has greater exposure to DRPs as the presence of such complex conditions is often associated with multiple drug therapy, hence, the patient comorbid condition and therapy should be carefully reviewed ensuring appropriate prescribing of medications and early prevention of DRPs.^42^

Antihypertensives were found to be the most commonly involved in DRPs in three studies with one identifying the ACE inhibitor drug class mainly involved with DRPs. Multiple drug therapy regimens including antihypertensives with other cardiovascular medications potentially reduces the effect of therapy and have greater exposure to adverse effects.^43^

Metformin demonstrates a significant involvement with DRPs in five studies.^20^^,^^23^^,^^30^^,^^31^^,^^34^ The DRPs involving metformin in these studies were mostly associated with DDIs with other non-antidiabetic drug classes including metoprolol, ranitidine, simvastatin, and ciprofloxacin. The mechanism of interaction between metformin with beta-blockers and H2 receptor blockers was mainly through inhibition of either organic cation transporters (OCT) or multidrug and toxin extruders (MATE) transporters or both. The reduction in metformin renal clearance and increase in metformin plasma concentration further increases the risk of developing metformin-associated lactic acidosis.^44^^,^^45^ Therefore, prescribers should be alert for patients with medications that have an effect on the OCTs and MATEs transporters.

Out of all twenty studies included, thirteen mentioned the risk factors contributing to DRPs. In all of the thirteen studies, a total of 23 risk factors were observed with the most prevalent being associated with the presence of comorbidities which increases number of medications reported in six studies with the majority having hypertension.^20^^,^^22^^,^^27^^,^^28^^,^^31^^,^^34^ The risk of developing hypertension in people with diabetes is higher as they often manifest insulin resistance compared to normal individuals.^46^ This was followed by the number of medications reported in four studies^22^^,^^27^, ^28^, 29. and polypharmacy in another four studies.^20^^,^^29^^,^^31^^,^^32^ Overall, the risk factor was mostly involved with the high number of prescribed medications resulting in higher exposure to DRPs.^47^ The exposure to DRPs can be reduced with the act of deprescribing (“a systematic approach to identify and discontinue medications in which potential harm outweighs the benefit and medications with unclear benefit”).^48^

## Strength and limitations

In the previous systematic reviews, the majority are focused on DRPs in the general inpatient diabetic population with a few on T2DM in inpatient and outpatient settings. This systematic review is one of the few that conducted reviews on the available literature on DRPs in hospitalized patients specifically with T2DM. Five electronic databases were used to search appropriate articles with various search strategies used in each database. The data on the most common drugs or drug classes involved with DRPs were limited. Several studies reported the name of common drugs involved only when it came to DDIs. The common drug or drug classes involved were stated without describing the DRPs it was associated with. Additionally, a few studies performed non-random sampling which may possibly generate highly biased data results. One of the studies that carried out a questionnaire study design based on patient perspective will have a risk of the individual not having the appropriate knowledge or recollection of the DRPs experienced to provide responses accurately affecting the results of the study.

From this systematic review, it can be observed that hospitalized T2DM patients have a high prevalence of DRPs with the most common being DDIs. Prescribers should be more cautious with this population, especially those who have co-morbidities as it increases the number of medications and risk of DDIs. The risk of DDIs could be reduced by regularly reviewing the patient list of medications and using a reliable source of information guidelines on common drug interaction with antidiabetics. Future studies on DRPs prevalence in hospitalized T2DM patients are necessary for further development of approaches in identifying, resolving, and preventing DPRs improving patient clinical outcomes.^49^ Recently, a new tool, The Alsayed_v1 tools, encompassing treatment assessments, medical-problem oriented plan, and care plan, have been validated and applied to real patient cases.^50^ This comprehensive tool can be modified for clinical pharmacists to efficiently identify, categorize, and address drug-related problems for T2DM.

## Conclusion

In this systematic review, the occurrence of DRPs in T2DM hospitalized patients was observed to be high with a diverse range among the included studies. DDIs were reported to be most prevalent in a majority of the studies. Antidiabetics (metformin) and antihypertensives showed a significant involvement with DRPs. The risk factors that were highly associated with DRPs were the presence of comorbidities (HTN), the number of medications and polypharmacy.

## Declaration of Competing Interest

The authors declare that they have no known competing financial interests or personal relationships that could have appeared to influence the work reported in this paper.

## Appendix A Supplementary data

Supplementary Information 1 PRISMA checklist. Supplementary material 1

Supplementary Information 3 Risk factors for drug-related problems Supplementary material 2

Supplementary Information 3 Quality assessment scores Supplementary material 3
