# Supplementary material for: Drug-related problems in hospitalized patients with type 2 diabetes mellitus: A systematic review
Source: Explor Res Clin Soc Pharm. 2023 Oct 12;12:100348. doi: 10.1016/j.rcsop.2023.100348 (PMC10598051; doi:10.1016/j.rcsop.2023.100348)
Supplement: Supplementary Information 3 — Risk factors for drug-related problems [file mmc2.pdf]

Article: Drug-related problems in hospitalized patients with type 2 diabetes mellitus: A systematic review
Journal: Exploratory Research in Clinical and Social Pharmacy; https://doi.org/10.1016/j.rcsop.2023.100348

Supplementary Information 2: Quality assessment scores

Studies	Q1: Was the sample frame appropriate to address the target population?	Q2: Were study participants recruited in an appropriate way?	Q3: Was the sample size adequate?	Q4: Were the study subjects and the setting described in detail?	Q5: Was the data analysis conducted with sufficient coverage of the identified sample?	Q6: Were valid methods used for the identification of the condition?	Q7: Was the condition measured reliably?	Q8: Was there appropriate statistical analysis?	Q9: Was the response rate adequate, and if not, was the low response rate managed appropriately?	Overall Appraisal (SCORE)
	Q1	Q2	Q3	Q4	Q5	Q6	Q7	Q8	Q9	Overall appraisal
Acharya et al., 2020 [1]	N	N	N	N	Y	N/A	U	Y	N/A	2
Corsonello et al., 2005 [2]	Y	Y	Y	Y	Y	Y	Y	Y	N/A	7
Herman et al., 2016 [3]	N	N (PURPOSIVE SAMPLING)	N/A	N	N	N/A	U	Y	N/A	1
Hidayati et al., 2018 [4]	Y	Y	N/A	Y	Y	N/A	U	Y	N/A	5
Hussain et al., 2019 [5]	Y	Y	N/A	Y	Y	N/A	U	Y	N/A	5
Inamdar et al., 2016 [6]	Y	Y	N/A	Y	Y	N/A	U	Y	N/A	5
Inamdar et al., 2020 [7]	Y	Y	N/A	Y	Y	N/A	U	N	N/A	4

Article: Drug-related problems in hospitalized patients with type 2 diabetes mellitus: A systematic review
Journal: Exploratory Research in Clinical and Social Pharmacy; https://doi.org/10.1016/j.rcsop.2023.100348

Indriani et al., 2010 [8]	Y	Y	N/A	Y	Y	N/A	U	Y	N/A	5
Indriani et al., 2019 [9]	Y	Y	N/A	Y	Y	N/A	U	N	N/A	4
Kosmalski et al., 2012 [10]	N	Y	Y	Y	Y	N/A	U	Y	N/A	5
Mader et al., 2022 [11]	Y	Y	N/A	Y	N	N/A	U	Y	N/A	4
Maharani et al., 2018 [12]	Y	Y	N/A	Y	Y	N/A	U	Y	N/A	5
Nigussie et al., 2022 [13]	Y	Y	N/A	Y	Y	N/A	U	Y	N/A	5
Nzayisenga et al., 2019 [14]	Y	Y	Y	Y	Y	N/A	U	Y	N/A	6
Okayasu et al., 2012 [15]	Y	Y	N/A	Y	Y	N/A	U	Y	N/A	5
Salam et al., 2018 [16]	Y	N (NOT RANDOM)	N/A	Y	Y	N/A	U	Y	N/A	4
Sharma et al., 2020 [17]	Y	Y	N/A	Y	Y	N/A	U	Y	N/A	5
Siregar et al., 2018 [18]	Y	N (PURPOSIVE SAMPLING)	N/A	Y	Y	N/A	U	Y	N/A	4
Varghese et al., 2007 [19]	Y	Y	N/A	Y	Y	N/A	U	Y	N/A	5
Zazuli et al., 2017 [20]	Y	Y	Y	Y	Y	N/A	U	Y	N/A	6
Abbreviations<br>Y: Yes; N: No; U: Unclear; NA: Not Applicable; I: Include; E: Exclude; S: Seek Further Information										

Article: Drug-related problems in hospitalized patients with type 2 diabetes mellitus: A systematic review
Journal: Exploratory Research in Clinical and Social Pharmacy; https://doi.org/10.1016/j.rcsop.2023.100348

References

1. Acharya Dr, L.D., *Study of Drug Related Problems in Type II Diabetes Mellitus patients with Comorbidities in a Tertiary Care Hospital-A Retrospective Study.* 2020.
2. Corsonello, A., et al., *Concealed renal failure and adverse drug reactions in older patients with type 2 diabetes mellitus.* Journals of Gerontology - Series A Biological Sciences and Medical Sciences, 2005. **60**(9): p. 1147-1151.
3. Herman, H., et al., *Inappropriate use of the drugto elderly patients with Type-II diabetes mellitus in Makassar Indonesia.* Der Pharmacia Lettre, 2016. **8**(3): p. 154-158.
4. Hidayati, N.R., P. Oktaviani, and I. Setyaningsih, *Gambaran Interaksi Obat Diabetes Melitus Tipe 2 Dengan Obat Penyakit Penyerta Pada Pasien Rawat Inap Di Rsud Gunung Jati Kota Cirebon Tahun 2016.* Medical Sains: Jurnal Ilmiah Kefarmasian, 2018. **2**(2): p. 58-64.
5. Hussain, M.A., S. Firdous, and M.E. Uz, *Role of clinical pharmacist in patients with diabetes and hypertension: a prospective study.* Role of clinical pharmacist in patients with diabetes and hypertension: a prospective study, 2019.
6. Inamdar, S. and R. Kulkarni, *Drug related problems in elderly patients with type 2 diabetes mellitus.* Journal of Diabetology, 2016. **7**(1): p. 1.
7. Inamdar, S.Z., R.V. Kulkarni, and V. Akhila, *Pharmacist Led Assessment of Drug Related Problems in Type 2 Diabetes Mellitus Patients.* 2020.
8. Indriani, D.V., *Evaluasi Drug Related Problems (DRPs) pada Pasien Diabetes Melitus Tipe 2 Non Komplikasi Di Instalasi Rawat Inap Rumah Sakit Panti Rini Yogyakarta Periode Januari 2009-Maret 2010*. 2010, Skripsi.
9. Indriani, L. and E. Oktaviani, *Drug Related Problems (DRPs) Identification on Diabetes Melitus Type 2 Ward Patients with Complication.* 2019.
10. Kosmalski, M., et al., *Inappropriate metformin prescribing in elderly type 2 diabetes mellitus (T2DM) patients.* Advances in Medical Sciences, 2012. **57**(1): p. 65-70.
11. Mader, J.K., et al., *Medication errors in type 2 diabetes from patients' perspective.* PLoS ONE, 2022. **17**(4 April).
12. Maharani, D.D., N.F. Syafhan, and Y. Hersunaryati, *Drug-related problems in hospitalized geriatric patients with diabetes mellitus.* International Journal of Applied Pharmaceutics, 2018. **10**(Special Issue 1): p. 142-147.
13. Nigussie, K.A., et al., *Prevalence and factors associated with inappropriate anti- diabetic medication therapy among type 2 diabetes mellitus patients at the medical and surgical wards of Mbarara Regional Referral Hospital, Uganda.* PLoS One, 2022. **17**(6): p. e0270108.
14. NZAYISENGA, J., *Drug-related problems among type II diabetes mellitus patients with hypertension: a cross-sectional study*. 2019, Mount Kenya University Rwanda.
15. Okayasu, S., et al., *The evaluation of risk factors associated with adverse drug reactions by metformin in type 2 diabetes mellitus*. Biological and Pharmaceutical Bulletin, 2012. **35**(6): p. 933-937.
16. Salam, N.S., *Identifikasi Drug Related Problems (DRPs) pada Pasien Diabetes Mellitus Tipe 2 dengan Komplikasi Penyakit Makrovaskular*. 2018, Universitas Islam Negeri Alauddin Makassar.
17. Sharma, R., et al., *Potentially Inappropriate Medication Use in Older Hospitalized Patients with Type 2 Diabetes: A Cross-Sectional Study.* Pharmacy (Basel), 2020. **8**(4).
18. Siregar, D.A., *Identification of Drug Related Problem (DRPs) in diabetes mellitus type 2 patients with hypertension at 5th floor of teratai in general hospital center (RSUP) Fatmawati Period January–June 2016*. Jakarta: Fakultas Ilmu Kesehatan UIN Syarif Hidayatullah.

19. Varghese, P., et al., *Hypoglycemia in hospitalized patients treated with antihyperglycemic agents.* Journal of Hospital Medicine, 2007. **2**(4): p. 234-240.

20. Zazuli, Z., A. Rohaya, and I.K. Adnyana, *Drug-related problems in Type 2 diabetic patients with hypertension in Cimahi, West Java, Indonesia: A prospective study.* International Journal of Green Pharmacy, 2017. **11**(2): p. S298-S304.
