# Supplementary material for: Drug-related problems in hospitalized patients with type 2 diabetes mellitus: A systematic review
Source: Explor Res Clin Soc Pharm. 2023 Oct 12;12:100348. doi: 10.1016/j.rcsop.2023.100348 (PMC10598051; doi:10.1016/j.rcsop.2023.100348)
Supplement: Supplementary Information 3 — Quality assessment scores [file mmc3.pdf]

Article: Drug-related problems in hospitalized patients with type 2 diabetes mellitus: A systematic review
Journal: Exploratory Research in Clinical and Social Pharmacy; https://doi.org/10.1016/j.rcsop.2023.100348

Supplementary Information 3: Risk factors for drug-related problems

Studies	Corsonello, et al. [1]	Hussain, et al. [2]	Inamdar and Kulkarni [3]	Inamdar, et al. [4]	Indriani and Oktaviani [5]	Mader, et al. [6]	Maharani, et al. [7]	Nigussie, et al. [8]	Nzayisenga [9]	Okayasu, et al. [10]	Salam [11]	Sharma, et al. [12]	Zazuli, et al. [13]	Total
Risk factors														
1. Concealed renal failure	✓													1
2. Presence of comorbidities (majority hypertension)		✓	✓	✓	✓			✓					✓	6
3. Presence of complications									✓		✓			2
4. Number of medications		✓			✓				✓ (>5)				✓	4
5. Lack of standard treatment protocol in the hospital		✓												1
6. Use of contraindicated drugs		✓												1
7. Elderly age > 60			✓				✓	✓						3
8. Age > 50					✓				✓					2
9. BMI > 25										✓				1
10. Polypharmacy			✓	✓					✓			✓		4

Article: Drug-related problems in hospitalized patients with type 2 diabetes mellitus: A systematic review
Journal: Exploratory Research in Clinical and Social Pharmacy; https://doi.org/10.1016/j.rcsop.2023.100348

11. Lack of appropriate information and knowledge on the pharmacokinetics				✓										1
12. Gender (female)					✓					✓		✓		3
13. Obesity					✓									1
14. Pregnancy					✓									1
15. Poor relationship and communication between doctor and patient						✓								1
16. Similarities in drug name and design						✓								1
17. Lack of medical staff						✓								1
18. T2DM duration > 10 years								✓						1
19. HbA1c < 7%								✓						1
20. Use of traditional medicine									✓					1
21. Elevated ALT and ALP										✓				1
22. Reduced renal function												✓		1
23. Length of stay													✓	1
Total	1	4	3	3	6	3	1	4	5	3	1	3	3	40

Article: Drug-related problems in hospitalized patients with type 2 diabetes mellitus: A systematic review
Journal: Exploratory Research in Clinical and Social Pharmacy; https://doi.org/10.1016/j.rcsop.2023.100348

References

1. Corsonello, A.; Pedone, C.; Corica, F.; Mazzei, B.; Di Iorio, A.; Carbonin, P.; Incalzi, R. A., Concealed renal failure and adverse drug reactions in older patients with type 2 diabetes mellitus. *Journals of Gerontology - Series A Biological Sciences and Medical Sciences* **2005,** 60, (9), 1147-1151.
2. Hussain, M. A.; Firdous, S.; Uz, M. E., Role of clinical pharmacist in patients with diabetes and hypertension: a prospective study. *Role of clinical pharmacist in patients with diabetes and hypertension: a prospective study* **2019**.
3. Inamdar, S.; Kulkarni, R., Drug related problems in elderly patients with type 2 diabetes mellitus. *Journal of Diabetology* **2016,** 7, (1), 1.
4. Inamdar, S. Z.; Kulkarni, R. V.; Akhila, V., Pharmacist Led Assessment of Drug Related Problems in Type 2 Diabetes Mellitus Patients. **2020**.
5. Indriani, L.; Oktaviani, E., Drug Related Problems (DRPs) Identification on Diabetes Melitus Type 2 Ward Patients with Complication. **2019**.
6. Mader, J. K.; Aberer, F.; Drechsler, K. S.; Pöttler, T.; Lichtenegger, K. M.; Köle, W.; Sendlhofer, G., Medication errors in type 2 diabetes from patients' perspective. *PLoS ONE* **2022,** 17, (4 April).
7. Maharani, D. D.; Syafhan, N. F.; Hersunaryati, Y., Drug-related problems in hospitalized geriatric patients with diabetes mellitus. *International Journal of Applied Pharmaceutics* **2018,** 10, (Special Issue 1), 142-147.
8. Nigussie, K. A.; Shegena, E. A.; Stephen, O. P.; Namugambe, J. S.; Yadesa, T. M., Prevalence and factors associated with inappropriate anti- diabetic medication therapy among type 2 diabetes mellitus patients at the medical and surgical wards of Mbarara Regional Referral Hospital, Uganda. *PLoS One* **2022,** 17, (6), e0270108.
9. Nzayisenga, J. Drug-related problems among type II diabetes mellitus patients with hypertension: a cross-sectional study. Mount Kenya University Rwanda, 2019.
10. Okayasu, S.; Kitaichi, K.; Hori, A.; Suwa, T.; Horikawa, Y.; Yamamoto, M.; Takeda, J.; Itoh, Y., The evaluation of risk factors associated with adverse drug reactions by metformin in type 2 diabetes mellitus. *Biological and Pharmaceutical Bulletin* **2012,** 35, (6), 933-937.
11. Salam, N. S. Identifikasi Drug Related Problems (DRPs) pada Pasien Diabetes Mellitus Tipe 2 dengan Komplikasi Penyakit Makrovaskular. Universitas Islam Negeri Alauddin Makassar, 2018.
12. Sharma, R.; Chhabra, M.; Vidyasagar, K.; Rashid, M.; Fialova, D.; Bhagavathula, A. S., Potentially Inappropriate Medication Use in Older Hospitalized Patients with Type 2 Diabetes: A Cross-Sectional Study. *Pharmacy (Basel)* **2020,** 8, (4).
13. Zazuli, Z.; Rohaya, A.; Adnyana, I. K., Drug-related problems in Type 2 diabetic patients with hypertension in Cimahi, West Java, Indonesia: A prospective study. *International Journal of Green Pharmacy* **2017,** 11, (2), S298-S304.
